# Effects of forest age and season on soil microbial communities in Chinese fir plantations

**DOI:** 10.1128/spectrum.04075-23

**Published:** 2024-07-09

**Authors:** Yuxin Hu, Xiongqing Zhang, Hanyue Chen, Yihang Jiang, Jianguo Zhang

**Affiliations:** 1 State Key Laboratory of Efficient Production of Forest Resources, Key Laboratory of Tree Breeding and Cultivation of the National Forestry and Grassland Administration, Research Institute of Forestry, Chinese Academy of Forestry, Beijing, China; 2 Collaborative Innovation Center of Sustainable Forestry in Southern China, Nanjing Forestry University, Nanjing, China; USDA-ARS-NPRL, Dawson, Georgia, USA

**Keywords:** Chinese fir plantations, soil microbial community, age-related changes, seasonal variations, high-throughput sequencing

## Abstract

Understanding changes in the distribution patterns and diversity of soil microbial communities from the perspectives of age-related changes, seasonal variations, and the interaction between the two factors can facilitate the management of plantations. In Chinese fir plantations, we collected soils from different depths in over-mature forests, mature forests, near-mature forests, middle-aged forests, and young forests in summer, autumn, and winter in China’s subtropical regions. As the forests developed, bacterial and fungal communities’ diversity changed, reached a minimum value at near-mature forests, and then increased in mature forests or over-mature forests. Near-mature forests had the lowest topological properties. The Shannon index of microbial communities varied with seasonal changes (*P* < 0.05). Bacterial and fungal community composition at genus level was more closely related to temperature indicators (including daily average temperature, daily maximum temperature, and daily minimum temperature) (*P* < 0.01, 0.5554 < *R*^2^ <0.8185) than daily average precipitation (*P* > 0.05, 0.0321 < *R*^2^ <0.6773). Bacteria were clustered by season and fungi were clustered by forest age. We suggested that extending the tree cultivation time of plantations could promote microbial community recovery. In addition, we found some species worthy of attention, including Bacteroidetes in autumn in over-mature forests, and Firmicutes in summer in young forests.

**IMPORTANCE:**

Chinese fir [*Cunninghamia lanceolata* (Lamb.) Hook] is an important fast-growing species with the largest artificial forest area in China, with the outstanding problems of low quality in soil. Soil microorganisms play a crucial role in soil fertility by decomposing organic matter, optimizing soil structure, and releasing essential nutrients for plant growth. In order to maintain healthy soil quality and prevent nutrient depletion and land degradation, it is crucial to understand the changes of soil microbial composition and diversity. Our study determined to reveal the change of soil microbial community from stand age, season, and the interaction between the two aspects, which is helpful to understand how interannual changes in different years and seasonal changes in one year affect soil fertility restoration and sustainable forest plantation management. It is a meaningful exploration of soil microbial communities and provides new information for further research.

## INTRODUCTION

Forest soil is indispensable for cultivating forests, providing essential nutrients and water for plant growth (1). Soil bacteria and fungi are crucial for preserving global biodiversity and biomass, contributing significantly to biogeochemical cycling (2, 3). Understanding the impact of soil management practices on soil quality, forest sustainability, and plant productivity relies heavily on soil microbiology (4). Predicting changes in microbial community distribution patterns and diversity can enhance our ability to anticipate alterations in plantation soil ecosystems, thereby facilitating improved measures for sustainable management (5). Bacterial and fungal communities both play crucial roles in regulating plant nutrition and soil organic matter cycling (6), essential for maintaining soil fertility and ecosystem functions (7, 8). In related research on forest soils, soil microbial community composition may respond differently to the variation in soil organic matter concentration and the imbalances nutrients in soil (9, 10). Bacteria are the most abundant soil microorganisms in forests. Increased soil nutrient availability and carbon cycling rate are associated with increased diversity of soil bacterial communities (11). The structure and diversity of bacterial communities may respond differently to soils in different ecosystems (12). Fungal communities differ significantly from bacterial communities in composition structure, distribution mode, and functional assumption (13, 14). Fungi play a crucial role in enhancing soil fertility and promoting plant growth during forest development, forming complex biodiversity assemblages strongly associated with high fertility levels (15, 16). Fungal diversity was significantly related to physical and chemical characteristics of soil, such as soil bulk density, texture, SOC, and TN, especially in the early succession stages (17). As primary decomposers and plant mutualists, soil fungi have more complex niches and form different functional groups that play different roles in nutrient cycling and carbon storage (18).

Investigating the response of soil microbial communities across various ages and seasons could yield valuable insights into their potential as indicators of soil quality (19–22). Soil microbial community compositions vary among forests of different ages, with the diversity and abundance of bacterial communities showing a temporal trend (23–25). During the course of forest development, bacterial and fungal communities exhibit distinct successional trajectories. The diversity of soil bacterial communities undergoes significant alterations primarily during the initial stages of this process. Conversely, the diversity within fungal communities was more sensitive to the forest development at the late stage (26). Soil fungal alpha diversity decreases with the aging of the ecosystem and the fungal beta diversity, microbial community composition also being influenced by stand age (27–30). Continuous cultivation of single-age monocultures might have negative feedbacks on the soil microbial communities (31), impacting soil carbon, nitrogen, and phosphorus cycling, as well as fungal and bacterial communities (32). Seasonal changes and succession exert a more complex influence on microbial diversity (33, 34). Seasonal changes have a decisive impact on soil microbial dynamics due to time-varying factors such as soil moisture, temperature, nutrient levels, and vegetation biomass (35). The comparative study indicated that biodiversity was influenced by distinct environmental predictors, and community morphotypes varied with season, temperature, and soil moisture (36). Changes in temperature and moisture conditions are associated with changes in microbial community composition or microbial metabolic diversity (37, 38). Microbial communities adapt to environmental changes by adjusting the ratio of dominant fungi, responding to moisture patterns, and altering the relationships between microbial communities and functional groups. Mean annual temperature influenced the dominant micro-organisms species (5) and mean annual precipitation and temperature were important factors affecting soil bacterial β-diversity (39, 40). Research on forests showed that soil moisture is the major factor influencing fungal community structure and the key variable associated with fungal community distributions (41, 42). However, increasing soil temperature raises the optimum for bacterial growth (43).

Chinese fir is an important tree species in China’s subtropical zone, used for forest cultivation, timber production, and ecological restoration. It is widely planted and can help reduce the exploitation of natural forests, improve the environment, and build an ecological civilization (44, 45). To properly manage Chinese fir plantations and restore ecological balance, it is crucial to understand the role of microbial communities in maintaining healthy soil quality and preventing nutrient depletion and land degradation. The objectives of this study are: (i) explore the changes in diversity, co-occurrence network structure, and composition distribution of bacterial and fungal communities at different forest ages; (ii) analyze the impacts of seasonal changes on diversity, composition distribution, and correlation between species composition and temperature and precipitation indicators of bacterial and fungal communities; and (iii) investigate the interaction impact of age and season on bacterial and fungal community diversity and composition.

## MATERIALS AND METHODS

### Study sites

The study area is located in *Shanxia* Forest Farm, *Fenyi* County, Jiangxi Province of China (27°30′N to 27°45′N and 114°30′E to 114°45′E). This region has a warm and humid subtropical monsoon climate, with the evergreen broad-leaved forest as the zonal plant. The mean annual temperature is 16.8°C, the annual precipitation is 1,600 mm, and the precipitation is mostly concentrated in spring. The site is mostly a low mountainous and hilly landscape. The soil is clay loam red soil (Alliti-Udic Ferrosols), according to I. IUSS Working Group, and the parent rocks are mostly shale. The meteorological data came from the daily hourly data records of the corresponding weather station in *Shanxia* Forest Farm in 2020–2021 (Table 1). The following were measured in each plot: location, elevation, slope, mean tree height, mean diameter at breast height (DBH), and density (Table 2).

**TABLE 1:** Basic climate information of the Chinese fir plantation in summer, autumn, and winter

	Average monthly temperature (°C)	Maximum monthly temperature (°C)	Monthly minimum temperature (°C)	Average monthly air humidity (%)	Average monthly precipitation (mm)
Summer	26.20	29.86	23.39	94.58	754.60
Autumn	22.29	26.25	18.87	91.95	265.50
Winter	7.09	23.27	−1.27	91.10	285.50

**TABLE 2:** Site characteristics of the Chinese fir plantation^*a*^

Type	Age (a)	Location	Elevation (m)	Slope (°)	Mean tree height (m)	Mean diameter at breast height (cm)	Density (trees/hm^2^)
Young forest (YOF)	8	114°39′36″E, 27°4424″N	107	20	7.37	10.11	3,333
		114°39′38″E, 27°44′26″N	115	24			
		114°39′32″E, 27°44′22″N	111	26			
Middle-aged forest (MIF)	14	114°39′29″E, 27°44′23″N	116	25	14.4	11.46	3,294
		114°39′27″E, 27°44′23″N	102	24			
		114°39′27″E, 27°44′20″N	103	30			
Near-mature forest (NMF)	20	114°39′15″E, 27°44′25″N	126	29	18.49	21.37	3,067
		114°39′26″E, 27°44′26″N	109	19			
		114°39′18″E, 27°44′22″N	119	21			
Mature forest (MAF)	27	114°39′32″E, 27°44′27″N	116	20	18.22	19.03	2,589
		114°39′30″E, 27°44′26″N	100	26			
		114°39′34″E, 27°44′26″N	108	28			
Over-mature forest (OMF)	51	114°39′31″E, 27°44′29″N	111	26	18.02	20.9	1,968
		114°39′28″E, 27°44′35″N	118	25			
		114°39′27″E, 27°44′35″N	119	30			

^*a*^ They are over-mature forest (51a), mature forest (27a), near-mature forest (20a), middle-aged forest (14a), and young forest (8a).

### Soil sampling

The plots were selected by the method of space-for-time substitution. Sampling with same site condition was performed during four different seasons: spring (4 April 2020), summer (23 July 2020), autumn (13 October 2020), and winter (17 January 2021). However, the samples collected in the spring were damaged during lab analysis. The samples collected in summer, autumn, and winter were used in this study and were labeled as A, B, and C, respectively. At the same time, the corresponding basic climate information is obtained (Tables 1 and 2). According to the age classification standard, the objects were overmature (51 years), mature (27 years), near-mature (20 years), middle-aged (14 years), and young (8 years) plantations, represented by over-mature forest (OMF), mature forest (MAF), near-mature forest (NMF), middle-aged forest (MIF), and young forest (YOF).

We selected three 20 m × 30 m sampling plots from each age. Three soil profiles were excavated in each plot. Soil samples in each soil profile were collected at three depths (*T*: 0–20 cm, *M*: 20–40 cm, and *B*: 40–60 cm). The litter and humus on the soil surface were removed before sampling. We sieved the samples through a 2-mm mesh to remove roots, rocks, plant tissues, and other items. The soil samples in each season were sent to the laboratory and frozen at −80°C for later genomic analysis.

### High-throughput sequencing

We used the HiPure Fast DNA Spin Kit for Soil (Magen, Guangzhou, China) to extract soil microbial DNA. The target region of the ribosomal RNA gene was amplified by PCR (95°C for 5 min, followed by 30 cycles at 95°C for 1 min, 60°C for 1 min, and 72°C for 1 min and a final extension at 72°C for 7 min), related PCR reagents were from New England Biolabs, USA. To amplify the V3-V4 region of the bacterial 16S rRNA gene, we used the following primers fused with unique barcodes: 341F (5′-CCTACGGGNGGGCWGCAG-3′) and 806R (5′-GGACTACHVGGGTATCTAAT-3′). To amplify the ITS2 region of fungal, the ITS3 KYO2 (5′-GATGAAGAACGYAGYRAA-3′) and ITS4 (5′-TCCTCCGCTTATTGATATGC-3′) were used (46, 47). We extracted and purified amplicon’s target region using the AxyPrep DNA Gel Extraction Kit (Axygen Biosciences, Union City, CA, USA). Purified amplicons were pooled in equimolar and paired-end sequenced (PE250) on an Illumina platform according to the standard protocols.

Raw reads were further filtered according to the following rules using FASTP (version 0.18.0) (48). Paired-end clean reads were merged as raw tags using FLASH (version 1.2.11) (49) with a minimum overlap of 10 bp and mismatch error rates of 2%, then noisy sequences of raw tags were filtered (50). The clean tags were clustered into operational taxonomic units (OTUs) of  ≥97% similarity using UPARSE (version 9.2.64) pipeline (51). The tag sequence with the highest abundance was selected as representative sequence within each cluster. The representative OTU sequences were classified into organisms by a naive Bayesian model using RDP classifier (version 2.2) (52) based on SILVA database (version 132) (53) for 16S and UNITE database (version 8.0) (54) for ITS. Chao1, Simpson, and Shannon indexes which were used to estimate the richness and evenness of the fungal and bacterial communities were calculated in QIIME (version 1.9.1) (55).

### Data analysis

Data analysis was performed in R (version 4.3.1) (56). Simpson, Shannon, and Good’s coverage indexes were used to estimate the richness and diversity of the fungal and bacterial communities in vegan package (57). Chao1 rarefaction curves were graphed to ensure adequate sampling and sequencing for analysis. After acquiring the statistical sequence feature table (OTU table), the data were normalized to derive the relative abundance of various species. Microbial species were chosen at different taxonomic levels, considering their relative abundance, and only those with a minimum abundance of 0.1% were retained to explore the differences. Two-way ANOVA of Alpha diversity indicators was performed in R to test the interaction of season and age. Visualization of the composition of different soil layers was done in the ggtern package (58). After normalizing the data, we used the heatmap package to draw the heatmap of different stages (59). Analyze the level of difference between seasons using circos package (60). Spearman correlation matrices of different ages were calculated in psych package (61). Relevant data with relative abundance (>0.1%) correlation at the genus level with an absolute value less than 0.5 and a significant *P* value greater than 0.05 were screened out, the value of the autocorrelation was converted, and then generate a matrix for network analysis. Transform the matrix into the adjacency list , construct a weighted undirected network, and the weight represents the Spearman correlation coefficient of the abundance using igraph package (62). Then manipulate and visualize the network in Gephi software, run and calculate the basic graphic parameters. Based on the selected data set, PCoA analysis and adonis test between different groups were calculated separately using the psych package (61). The Canonical Correspondence analysis test was used to explore the relationships between environmental variables of different seasons in vegan package (57). Afterward, a permutation test based on 999 permutations was done, *P* value and *R*^2^ were obtained. Except for the co-occurrence network, the analysis in this paper used ggplot2 for visualization after extracting the related data (58).

## RESULTS

### Differences in soil microbial communities in the stands with different layers

The sampling process provided good coverage across different soil layers for various microbial communities (Fig. S1). Alpha diversity index and OTU numbers showed no significant differences among soil layers (*P* > 0.05) (Fig. S2). Regarding bacterial communities, species richness, evenness, Shannon diversity, Simpson diversity, and OTU number exhibited a “high-low-high” pattern, with the lowest values in the middle layer. Conversely, no significant trends were observed in these indicators for fungal communities.

**Fig 1:**
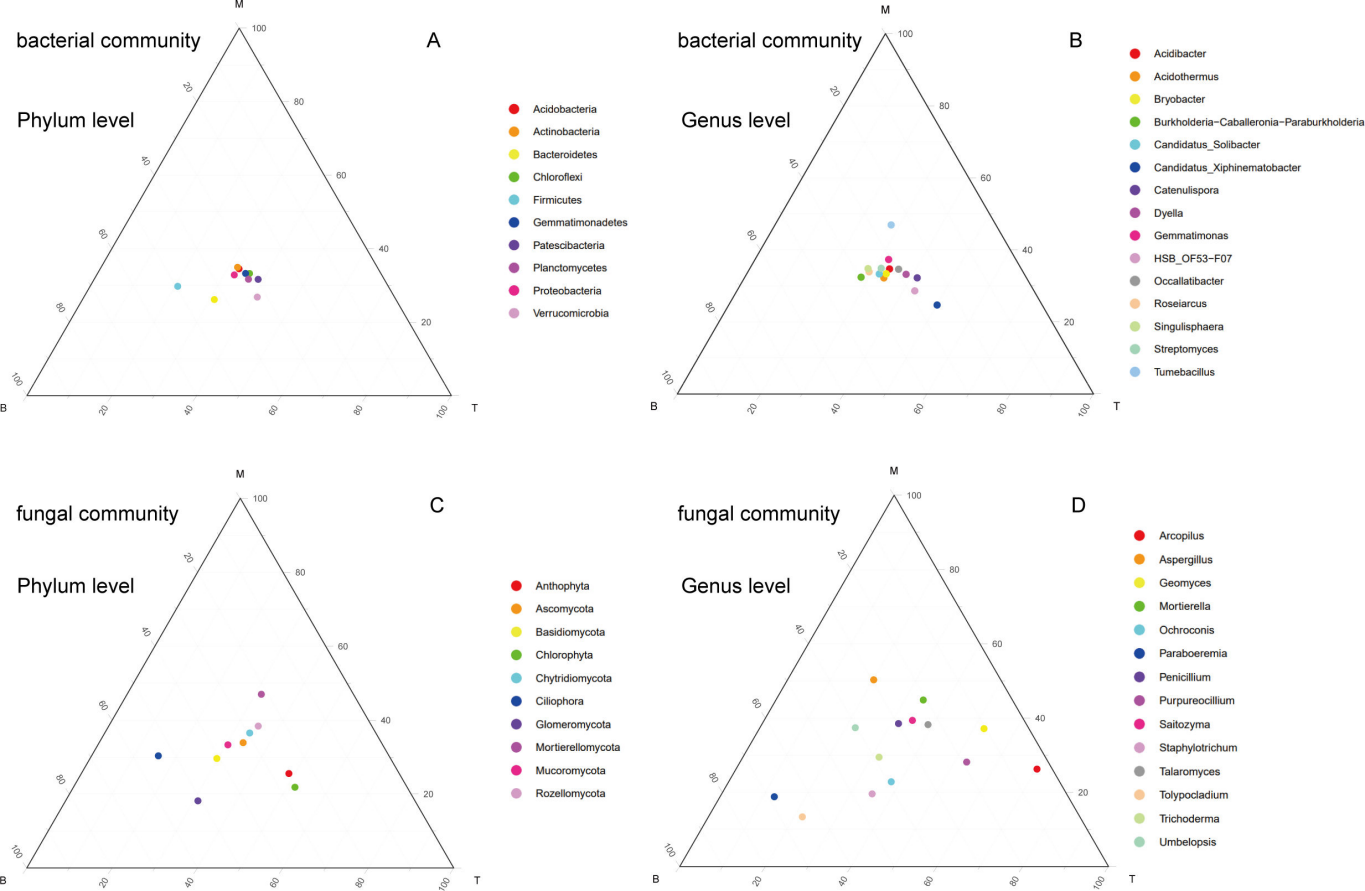
Ternary plots of species composition at different taxonomic levels of different forest layers. The three coordinate axes represent three different soil layers, respectively (*T*: 0–20 cm, *M*: 20–40 cm, and *B*: 40–60 cm). The order of species in the legend is ranked from largest to smallest according to the relative abundance of species in the community. The four figures are: (**A**) bacterial community at phylum level, (**B**) bacterial community at genus level, (**C**) fungal community at phylum level, and (**D**) fungal community at genus level

In soil bacterial communities, Firmicutes showed higher abundance in the M layer at the phylum level (Fig. 1A). At the genus level, *Tumebacillus* and *Candidatus_Xiphinematobacter* were relatively abundant in the B and T layers, respectively (Fig. 1B). As for soil fungal communities, Chlorophyta exhibited higher abundance in the B layer at the phylum level (Fig. 1C). At the genus level, significant variations were observed among species across different layers. *Paraboeremia* and *Tolypocladium* had a distribution proportion of over 50% in the M layers. While *Arcopilus* accounted for over half of the relative abundance in the B layer (Fig. 1D).

### Differences in soil microbial communities in the stands with different ages

**Fig 2:**
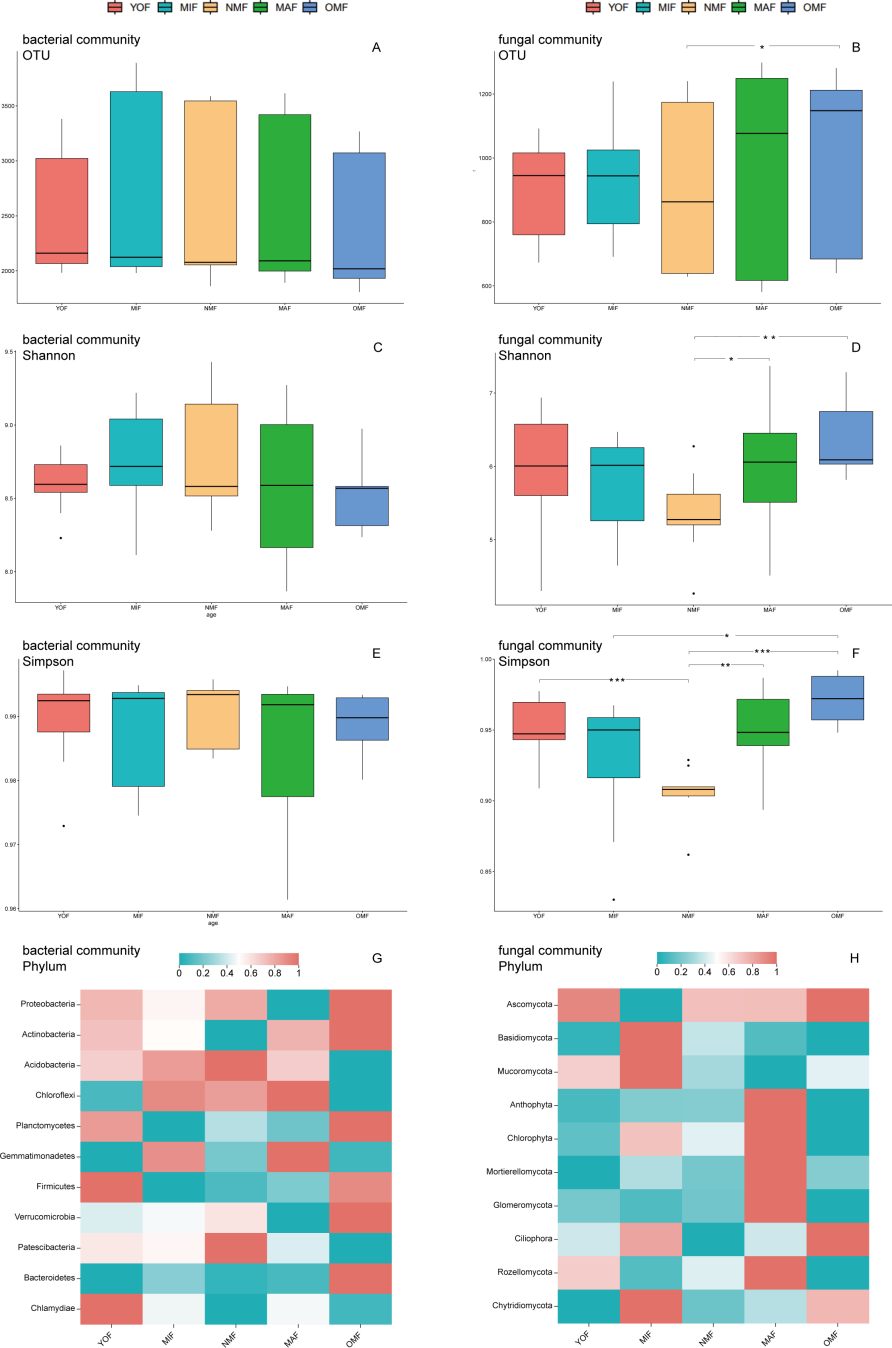
(**A and B**) Analysis of OTU numbers, (**C–F**) Alpha diversity index (Shannon and Simpson indices) of microbial communities in different forest ages. (**G and H**) Composition of microbial communities in different forest ages, different colors represent the relative abundance after normalizing the data. Red represents a large relative abundance, blue represents a small relative abundance, and the depth of the color represents the degree (OMF = over-mature forest, MAF = mature forest, NMF = near-mature forest, MIF = middle-aged forest, YOF = young forest, * =*P* < 0.05, ** =*P* < 0.01, *** =*P* < 0.001).

Significant differences in Sobs, Chao1, and ACE were observed among bacterial and fungal communities of different ages (*P* < 0.05) (Table 3). The Shannon and Simpson indices for bacterial communities showed slight fluctuations, initially increasing and then decreasing (Fig. 2C and E). Analyzing the Shannon and Simpson indices of fungal communities, significant increases were observed compared to MIF, with MAF and OMF showing significant differences (*P* < 0.05) (Fig. 2D and F).

**TABLE 3:** Two-way ANOVA of alpha diversity indicators in bacterial and fungal communities (* =*P* < 0.05, ** =*P* < 0.01, *** =*P* < 0.001)

Index	Community	Indicator	*F*	Significance
Age	Bacterial community	Sobs	8.71	8.66E−05***
		Shannon	2.20	0.09
		Simpson	15.56	5.37E−07***
		Chao1	6.63	4.00E−04***
		ACE	5.52	1.88E−03***
	Fungal community	Sobs	5.93	1.12E−03***
		Shannon	4.07	0.01**
		Simpson	1.61	0.20
		Chao1	6.10	1.03E−03***
		ACE	5.98	1.16E−03***
Season	Bacterial community	Sobs	886.72	2.07E−27***
		Shannon	39.63	3.80E−09***
		Simpson	6.98	3.24E−03***
		Chao1	1470.85	1.15E−30***
		ACE	1374.61	3.15E−30***
	Fungal community	Sobs	473.47	2.04E−23***
		Shannon	11.01	2.59E−04***
		Simpson	1.26	0.30
		Chao1	376.68	5.59E−22***
		ACE	457.45	3.36E−23***
Age * season	Bacterial community	Sobs	4.20	1.80E−03***
		Shannon	3.32	0.01**
		Simpson	5.60	2.22E−04***
		Chao1	3.74	3.83E−03***
		ACE	3.93	2.79E−03***
	Fungal community	Sobs	10.54	6.72E−07***
		Shannon	0.95	0.49
		Simpson	3.19	0.01**
		Chao1	10.28	8.76E−07***
		ACE	14.00	3.16E−08***

**Fig 3:**
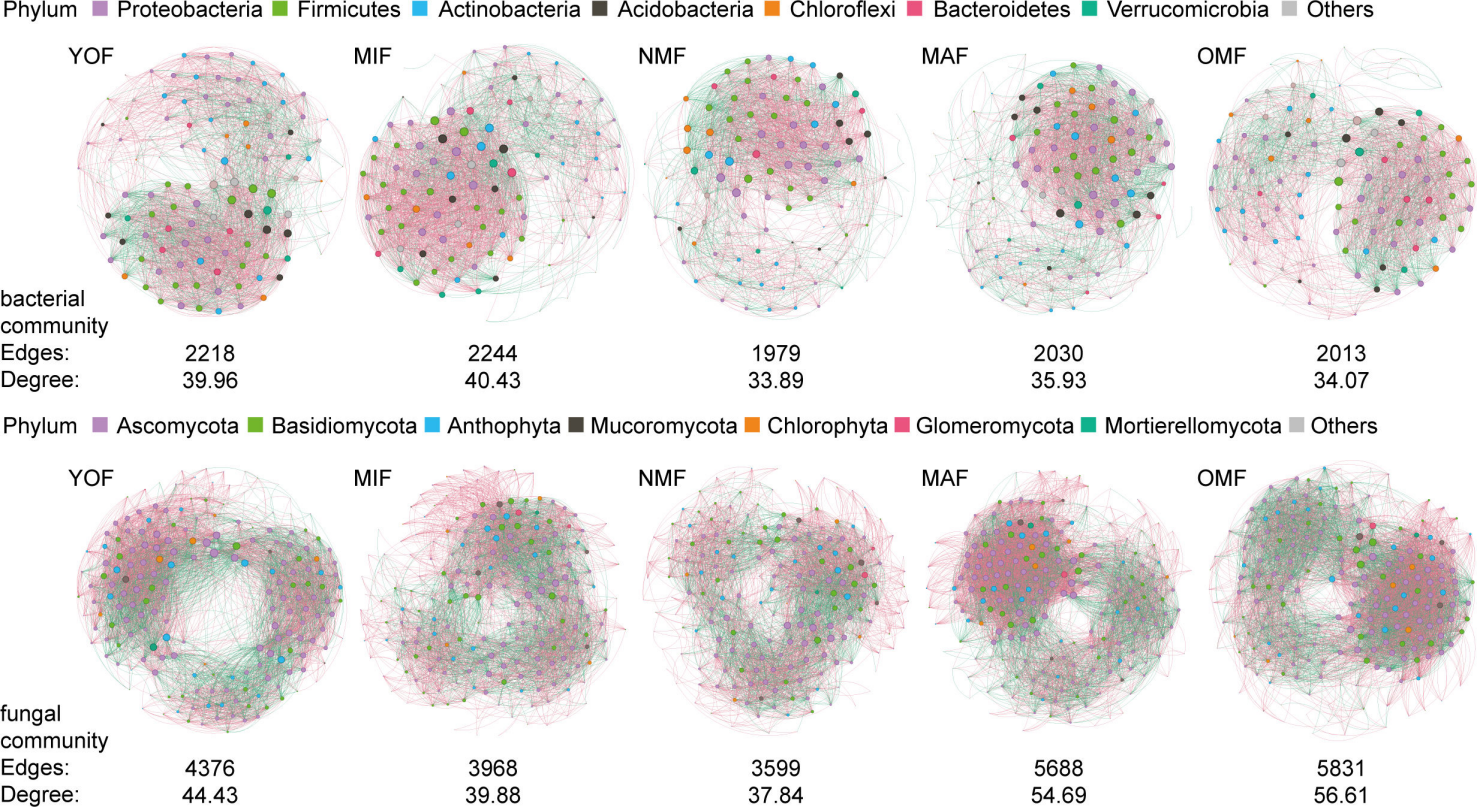
Co-occurrence networks of the bacteria community and fungi community in different forest ages. The nodes represented unique genus, the size of each node is proportional to the degree and each color indicates the different phylum. A connection stood for a strong (Spearman’s absolute coefficient >0.6) or significant (*P* < 0.05) correlation. The red line represented a positive correlation and the green line represented a negative correlation.

Moreover, the number of bacterial OTUs exhibited a decreasing trend from YOF to OMF (Fig. 2A), while fungal communities showed the fewest OTUs in NMF and the highest in OMF (Fig. 2B). In bacterial community, Proteobacteria, Actinobacteria, Bacteroidetes, Verrucomicrobia, and Planctomycetes were more abundant in OMF, with Patescibacteria being predominant in NMF (Fig. 2G). Regarding fungal communities, Anthophyta, Chlorophyta, Mortierellomycota, Glomeromycota, and Rozellomycota were relatively abundant (Fig. 2H). Phylum-level species composition changed with stand age. As age increased, the edges and average degrees of the co-occurrence networks of bacterial and fungal communities displayed a “high-low-high” pattern, suggesting that microbial communities in NMF exhibited lower complex topological properties compared to YOF and OMF (Fig. 3).

### Differences in soil microbial communities in the stands with different seasons

**Fig 4:**
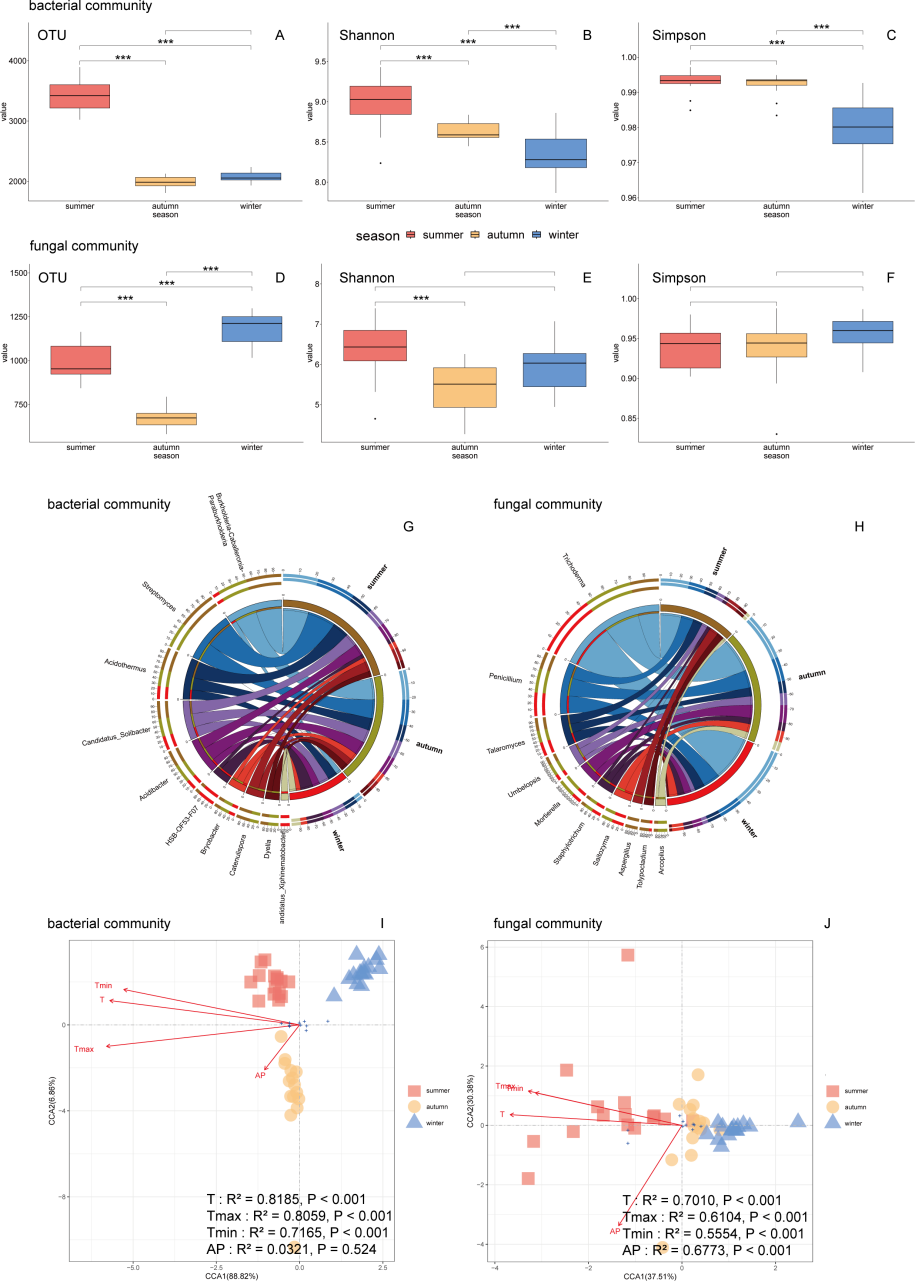
(**A and D**) Analysis of OTU numbers, (**B, C, D, F**) Alpha diversity, and (**G and H**) composition of microbial communities in different seasons. Shonnon and Simpson indices were chosen to study the relationship between Alpha diversity among communities in (**B, C, D, F**). (**I and J**) Canonical correspondence analysis (CCA) of the relationship between species diversity and climate in different seasons. The larger the *R*^2^ value, the higher the correlation between environmental factors and bacterial community structure. We use the Envfit test to calculate the *P* value of each environmental factor. The *P* value is used to determine whether the impact of the environmental factor is significant (* =*P* < 0.05, ** =*P* < 0.01, *** =*P* < 0.001).

Significant differences were observed in all bacterial community indicators and all indicators except Simpson’s index in fungal communities across different seasons (*P* < 0.05) (Table 3). Alpha indices indicated higher bacterial and fungal species diversity and evenness during summer (Fig. 4B, C, E, and F). The Shannon index of microbial communities decreased with seasonal changes (*P* < 0.05). The highest OTU numbers were observed in summer for bacterial communities and winter for fungal communities (Fig. 4A and D).

In bacterial communities, the dominant species and rankings remained similar between summer and autumn, in summer, at the genus level, *Streptomyces*, *Burkholderia-Caballeronia-Paraburkholderia*, and *Acidothermus* were the top three species. In autumn, *Burkholderia-Caballeronia-Paraburkholderia*, *Streptomyces*, *Acidibacter*, and *Candidatus_Solibacter* accounted for over 50% of the relative abundance. In winter, there was a different species distribution. *HSB_OF53-F07*, *Candidatus_Solibacter*, and *Acidothermus* replaced species with high abundance in the previous seasons (Fig. 4G).

Regarding fungal communities, Trichoderma, Penicillium, and Talaromyces exhibited higher abundances at the genus level during summer, with Trichoderma being the most abundant genus in winter (Fig. 4H). The bacterial and fungal community compositions showed stronger correlations with daily average temperature, daily maximum temperature, and daily minimum temperature (*P* < 0.01, 0.5554 < *R*^2^ <0.8185) than with daily average precipitation (*P* > 0.05, 0.0321 < *R*^2^ <0.6773). Specifically, in summer, the average temperature and the highest temperature were positively related to microbial communities, whereas in winter, a negative correlation was observed (Fig. 4I and J).

### Differences in soil microbial communities in the stands with different ages and seasons

**Fig 5:**
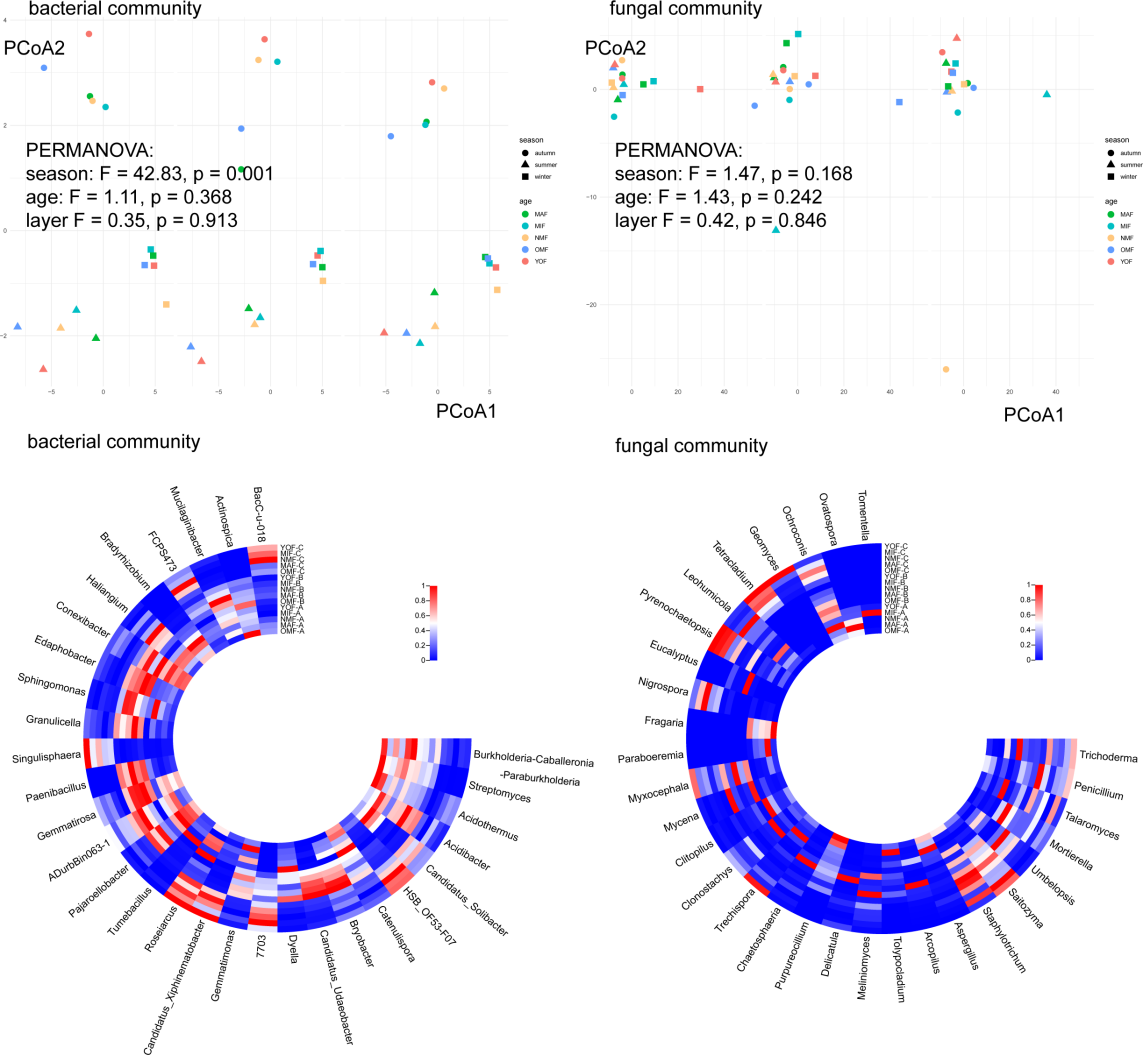
PCoA analysis and heatmap of soil microbial communities in different forest age stands in different seasons at genus level. In the PCoA analysis chart, different shapes represent different seasons, and different colors represent different forest ages. The *F* value and *P* value are obtained through the PERMANOVA test. The *F* value is used to measure the ratio of the difference between groups to the difference within the group. The larger the *F* value, the greater the difference within the group relative to the difference within the group. The heatmap is drawn based on normalized data. The darker the red, the greater the relative abundance in the corresponding sample, and the darker the blue, the smaller the relative abundance (OMF = over-mature forest, MAF = mature forest, NMF = near-mature forest, MIF = middle-aged forest, YOF = young forest; A = summer, B = autumn, C = winter).

After conducting PCoA analysis, PERMANOVA tests were performed to examine the *F*-values across different communities. In the bacterial community, the *F*-value order was season > age > layer, while in the fungal community, it was age > season > layer. Bacterial communities varied significantly between seasons, whereas fungal communities varied notably between ages (*P* < 0.05). No differences were observed between different soil layers in both communities (Fig. 5). Considering the interactions between age and season, significant differences were found in all bacterial community indicators and indicators except Shannon’s index in fungal communities between different seasons and ages (*P* < 0.05) (Table 3). In the bacterial communities, during summer, the species abundance of MIF was the highest, while in winter, YOF exhibited the highest species richness. For fungal communities, during summer and winter, MAF had the highest species richness, OMF had the highest evenness, and NMF had the lowest evenness (Fig. S3).

Observing specific seasons and forest age groupings, certain species showed high relative abundance at the genus level, possibly influenced by the interaction between forest age and season. In the bacterial community, at the genus level, *Burkholderia-Caballeronia-Paraburkholderia*, *Actinospica*; *Conexibacter*, *Edaphobacter*, *Gemmatiorosa* were highly abundant in OMF-A and OMF-B. *Tumebacillus*, *Catenulispora*; *BacC-u-018*, *Haliangium*, *ADurbBin063-1*, *Pajaroellobacter*, *Candidatus_Udaeobacter*; *Singulisphaera*, *Candidatus_Xiphinematobacter*, and *Roseiarcus* had high abundance in YOF-A, YOF-B, and YOF-C, respectively (Fig. 5). In the fungal community, in YOF, *Clitopilus*, *Talaromyces*; *Mycena*, *Staphylotrichun*; *Geomyces*, *Pyrenochaetopsis*, *Trechispora* showed high relative abundance in summer, autumn, and winter. In OMF, *Fragaria*, *Purpureocillium*; *Mycena*, *Clonostachys*, *Trichoderma*; *Staphylotrichun* were enriched in summer, autumn, and winter (Fig. 5).

## DISCUSSION

### Differences among layers of soil microbial communities

In the bacterial community, the diversity of the bacterial community in the middle layer is low, and the diversity of the top layer is the highest. However, no obvious regular trend was shown in the fungal community (Fig. S2). One possible reason is that soil depth primarily shaped bacterial communities, while plant species structure influenced fungi in the forests (63). Former studies showed that the predicted bacterial diversity in the topsoil was higher than in the corresponding subsoil (64). Compared with surface soils, the amount of bacterial biomass was much lower in deeper soils and microbial turnover was significantly slower (65–68).

In our study, the fungal species composition exhibited significant variation across soil depths, whereas the bacterial community composition remained relatively constant (Fig. 1). The predominant soil acidity in the study area is attributed to the autotoxicity of organic acids and phenolic compounds exuded by Chinese fir, in addition to the escalating impact of acid rain pollution. As a result, the acidity of the soil is been further aggravated (69). Therefore, the soil pH value of fir plantations is low. Previous research has indicated fungal dominance in acidic soil conditions and the topsoil exhibits a higher prevalence of fungi compared to bacteria and actinomycetes in our study, attributed to the lower soil pH (70, 71). Fungal have close relationships with plant rhizosphere and their interactions with roots at different depths may facilitate diverse life strategies among fungal taxa. Soil fungi in deeper layers of the soil profile contribute to carbon and nutrient cycling, soil formation, and xenobiotic degradation, which will improve water and nutrient absorption (72, 73). Based on the above viewpoints, it is necessary to analyze the changes in fungal communities. And the distribution of *Aspergillus*, which plays a crucial role in the natural process of making phosphorus accessible to plants, exhibited specificity in the soil at depths of 0–20 cm (74–76).

### Effects of different ages on soil microbial communities

Stand age is a key factor in evaluating soil biomass dynamics, carbon storage, contributing diversity, and other ecological processes, which may lead to changes in the diversity of soil microbial communities (77). The structure of microbial communities is influenced by stand age, and soil physical and chemical properties. Our research on co-occurrence networks has also shown that forest soil microbial connectivity and complexity were restored with age (Fig. 3). In general, soil microbial biomass or community diversity is traditionally used as an indicator of soil fertility (78, 79). Our study found that older forests had a higher species richness, diversity, and OTU in both soil microbial communities, especially the fungal. Soil microbial communities changed with forest development, it showed a high-low-high pattern (Fig. 2). In the Chinese fir plantation, microbial biomass and diversity were highest in YOF, OMF, or MAF, but there was a lack of homogeneity in YOF. Soil microbial communities in YOF differ significantly from soils in older plantation plots (74, 80). Cao et al. (81) showed that both the microbial diversity index and OTU increased with the increase in the forest age. The possible reason is that the artificial forest needs to carry out management measures such as afforestation and land preparation at the beginning of planting (82). In our study, the bacterial community gradually changed to a K-strategy, while the fungal community prioritized quality and quantity in their reproduction (83). Therefore, an appropriate extension of the plantation’s cultivation time is conducive to the restoration of soil properties and microbial communities to improve soil quality.

Our study showed that the dominant bacterial phyla at all ages were Actinobacteria, Proteobacteria, and Acidobacteria, while the dominant fungal phyla were Ascomycota and Basidiomycota, these results were confirmed by other studies of changes in Chinese fir plantations (20, 74, 84). The relative abundance of dominant species in bacterial communities and fungal communities in different forest stages is similar, probably because these dominant organisms have wide ranges of tolerance and resource utilization capabilities (85). After studying the bacterial community of Chinese fir plantations between 3 and 26 years old, it was found that the relative abundance of soil Proteobacteria first elevated and afterward reduced, while soil Acidobacteria increased over time. The above results are the same as those for YOF to MAF stages of forest growth in our study (83).

### Effects of different seasons and climates on soil microbial communities

Under the influence of a subtropical monsoon climate, environmental variability exhibits pronounced year-to-year fluctuations, driven by seasonal alterations in environmental conditions. Seasonal changes in climatic conditions play a key role in shaping soil microbial community dynamics, with direct effects on microbial communities through soil moisture and temperature (86–89). Temporal patterns of microbial growth and nutrient fixation-release cycles often reflect seasonal changes, and this response varies depending on the ecosystem’s specific moisture and temperature regimes (88). Previous research on forest soils has suggested that environmental factors had a more significant effect on microbial diversity than plant diversity. MAT and MAP explained most of the variations in the compositions of soil microbial communities (90). Analysis showed that temperature and moisture content were associated with 20% of the variability in soil microbial structure (91). Redundancy analysis demonstrates that environmental factors such as water content, organic matter, available phosphorus, and available potassium significantly influence soil microbial communities in Chinese fir plantations.

Strong associations exist between the microbial communities of Chinese fir and prevailing environmental conditions. Bacteria and fungi respond differently to seasonal environmental factors (87). Our analysis of microbial community diversity across seasons revealed substantial variation in both species diversity and composition within the bacterial community, with peak diversity indices observed during summer. The average and maximum temperature had a more significant effect on bacterial community, but all elements investigated including AP had a significant effect on fungal community (Fig. 4). Soil temperature was the main factor influencing differences in microbial community structure, bacterial community composition was mostly driven by temperature rather than other environmental factors, and the community diversity and distribution were regulated by the interaction and comprehensive regulation of various environments (92, 93). The reason for the highest bacterial community diversity in summer may be that the temperature rises weakened species interactions, in particular, the combination of increased precipitation and warming significantly increases the bacterial richness and decreases fungal richness (94). Rainfall seasonality is the main factor related to the structure and function of tropical forest soil bacterial communities, especially factors related to moisture content (95, 96). Compared with fungi, the composition and growth of bacterial communities are significantly affected by atmospheric humidity. Fungi are generally considered to be more drought-tolerant than bacteria, drought decreased alpha diversity and proportion of the total biomass of bacteria (97–99). Therefore, the research on the effects of different seasons and climates on soil microbial communities helps to understand the mechanism of soil fertility changes and then provides theoretical support and prediction basis for future management measures.

### Differences among ages and seasons from the perspective of species composition

Results of species composition showed differences when considering the interaction between seasons and ages, as compared to separate factors. Bacterial community species composition exhibited similar characteristics in the same season, while fungal communities tended to cluster by stand age (Fig. 5). Fungi have an important role in soil ecology by cycling nutrients and carbon, supporting plant nutrition and protection, and contributing to the diversity of pathogens (100). Fungal guilds are key integrators of plant richness-stock relationships, with fungal growth dominating the forest soil (101–103).

In our study, Proteobacteria, Actinobacteria, and Acidobacteria were the most common phyla in bacterial communities during summer and winter, with significant differences between these two seasons (Fig. S4). In similar studies, Proteobacteria, Acidobacteria, and Actinobacteria were the most prevalent soil bacteria in South China (81, 104, 105).Microbial communities of bacterial classification exhibit the most prominent features after the interaction, regarding specific functions. Proteobacteria fix nitrogen, alleviate soil phosphorus limitations, increase bacterial diversity, stimulate microbial groups, and prompt lipopolysaccharide biosynthesis and carbohydrate metabolism (106, 107). Acidobacteria and Proteobacteria were most affected by land-use change and were the most abundant taxonomic groups of soil bacteria (108, 109). They were more abundant in summer and in young and over-mature forests, which corresponded to high bacterial alpha diversity in our study. Acidobacteria had oligotrophic nature or ecological K-strategy (110). They decompose organic matter, recycle nutrients, regulate biogeochemical cycles, decompose biopolymers, and promote plant growth. The biofertilizer increases nutrients by Acidobacterial inoculation (111). Future research could explore initiatives aimed at manipulating crop rhizosphere with Acidobacterial populations to increase plant growth (112). They were more abundant in autumn, especially in young forests, where bacterial species richness was high and the within-group difference was small, indicating good uniformity. Actinobacteria produce beneficial metabolites such as antibiotics, biopolymers, and biocatalysts. Actinobacteria have an important influence on the turnover of recalcitrant plant organic matter in rhizosphere microbial communities. The rhizosphere region is considered one of the best habitats for isolating these microbes (112, 113). In the soil, Bacteroidetes are mattered with complex organic matter, especially the polysaccharides and proteins (114). And Bacteroidetes in the soil secrete diverse arrays of CAZymes which target the highly varied glycans (115). In soil, Firmicutes species possess iron and sulfate reduction abilities and have a critical role in soil disease control (107). Members of the Firmicutes group which have iron and sulfate-reducing abilities can be developed as effective bioenhancers in future bioremediation applications (116). In the forest soil, a potential function of *Arcopilus* in the environment is bioremediation of soils contaminated with organic matter and abnormal pH.

In the plantations, extending the planting period of plantations appropriately could help maintain the diversity of soil bacterial and fungal communities and improve soil quality (117). According to our study, microbial community diversity in plantations increased when forests are mature or over-mature, which indicated that we should pay more attention to the management of cultivation time to achieve the goal of soil fertility maintenance.

### Conclusions

We found that soil bacterial and fungal communities diversity changed with stand age, reached a minimum value at near-mature forest, and then increased. We suggested that extending the tree cultivation time of plantations could promote microbial community recovery. Considering differences of composition structure, the characteristics of bacterial communities in the same season were similar, while the fungal communities in the same stand age were similar. Both communities were significantly correlated with mean temperature, maximum and minimum temperature, and the fungal community was also affected by changes in precipitation (*P* < 0.05).

Considering the increasing diversity, richness, connectivity, and complexity of fungal community, it recovered after forest matured. We suggested that fungi will become a more important indicator of soil fertility and play a predominate role in the development of soil ecosystems in long-term cultivated plantations.

In the bacterial community, it had the highest richness and diversity in summer. Proteobacteria, Actinobacteria, and Acidobacteria were the most common phyla in all stand ages during summer and winter. In over-mature forest, the species composition was characterized by Actinobacteria in the summer and Bacteroidetes in the autumn. In the young forest, Firmicutes was dominant in the summer, potentially be of reference value for future research. These species may be key species for soil fertility restoration.

## Data Availability

The data sets generated during and/or analyzed during the current study are available from the corresponding author on reasonable request. All metagenomic raw reads used in this study have been deposited in the NCBI SRA database under BioProject IDs PRJNA1122241 and PRJNA1122874.

## SUPPLEMENTAL MATERIAL

Supplemental material spectrum.04075-23-s0001.docx Fig. S1 to S4.

ASM does not own the copyrights to Supplemental Material that may be linked to, or accessed through, an article. The authors have granted ASM a non-exclusive, world-wide license to publish the Supplemental Material files. Please contact the corresponding author directly for reuse.
