# Supplementary material for: Effects of forest age and season on soil microbial communities in Chinese fir plantations
Source: Microbiol Spectr. 2024 Jul 9;12(8):e04075-23. doi: 10.1128/spectrum.04075-23 (PMC11302042; doi:10.1128/spectrum.04075-23)
Supplement: Supplemental material — Fig. S1 to S4. [file spectrum.04075-23-s0001.docx]

**Supplementary materials**

# Effects of forest age and season on soil microbial communities in Chinese fir plantations

Yuxin Hu ^1,2^, Xiongqing Zhang^1,2,^ *, Hanyue Chen^1^, Yihang Jiang^1^, Jianguo Zhang^1^

1 State Key Laboratory of Efficient Production of Forest Resources, Key Laboratory of Tree Breeding and Cultivation of the National Forestry and Grassland Administration, Research Institute of Forestry, Chinese Academy of Forestry, Beijing 100091, China

2 Collaborative Innovation Center of Sustainable Forestry in Southern China, Nanjing Forestry University, Nanjing, 210037, P. R. China

*Corresponding author: Xiongqing Zhang

Email: xqzhang85@caf.ac.cn. Tel: 0086-10-62888309. Fax: 86-10-62872015.


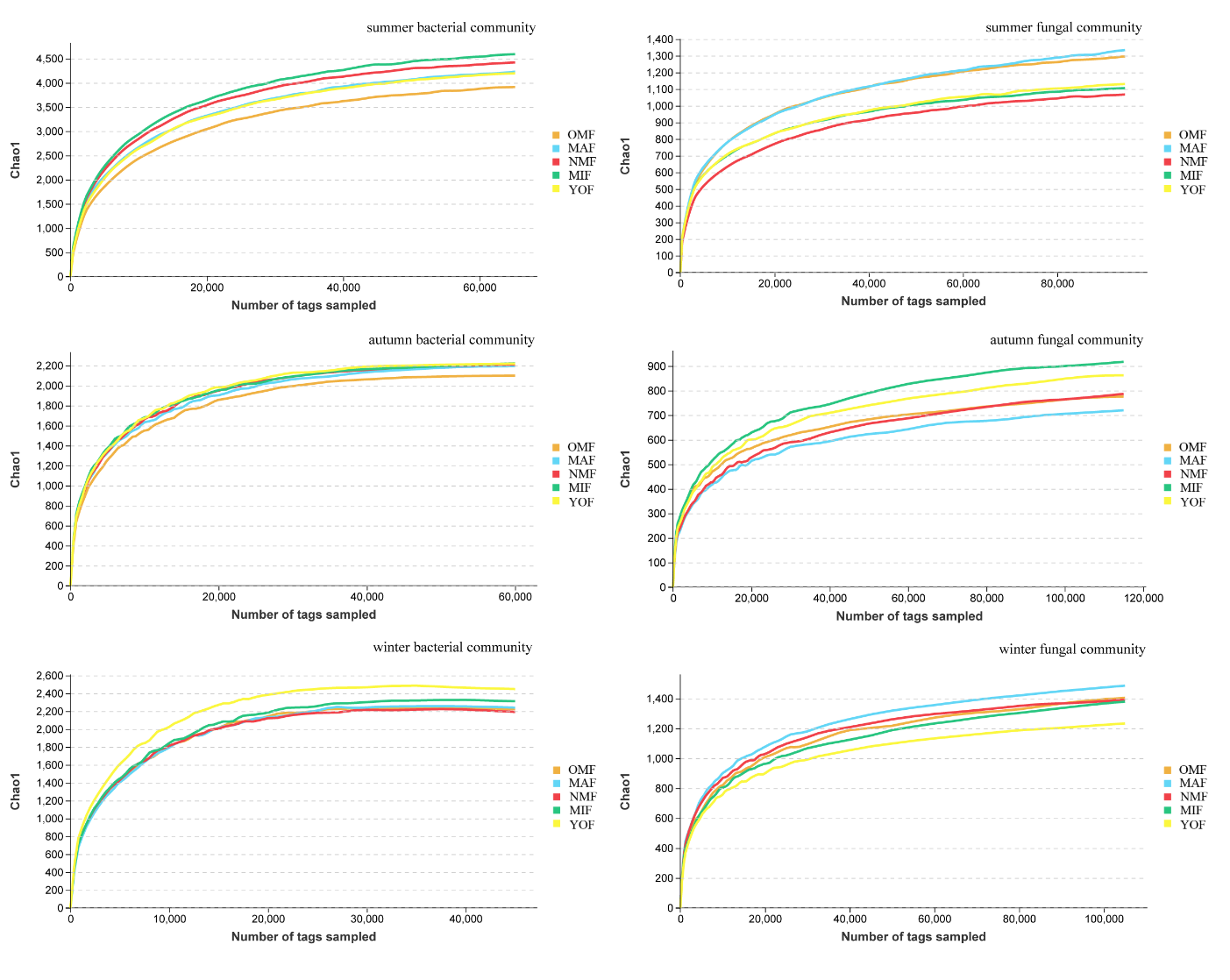


**Fig.S1** Rarefaction curves of Chao1 of the microbial community in different seasons. (OMF = over-mature forest, MAF = mature forest, NMF = near-mature forest, MIF = middle-aged forest, YOF = young forest).


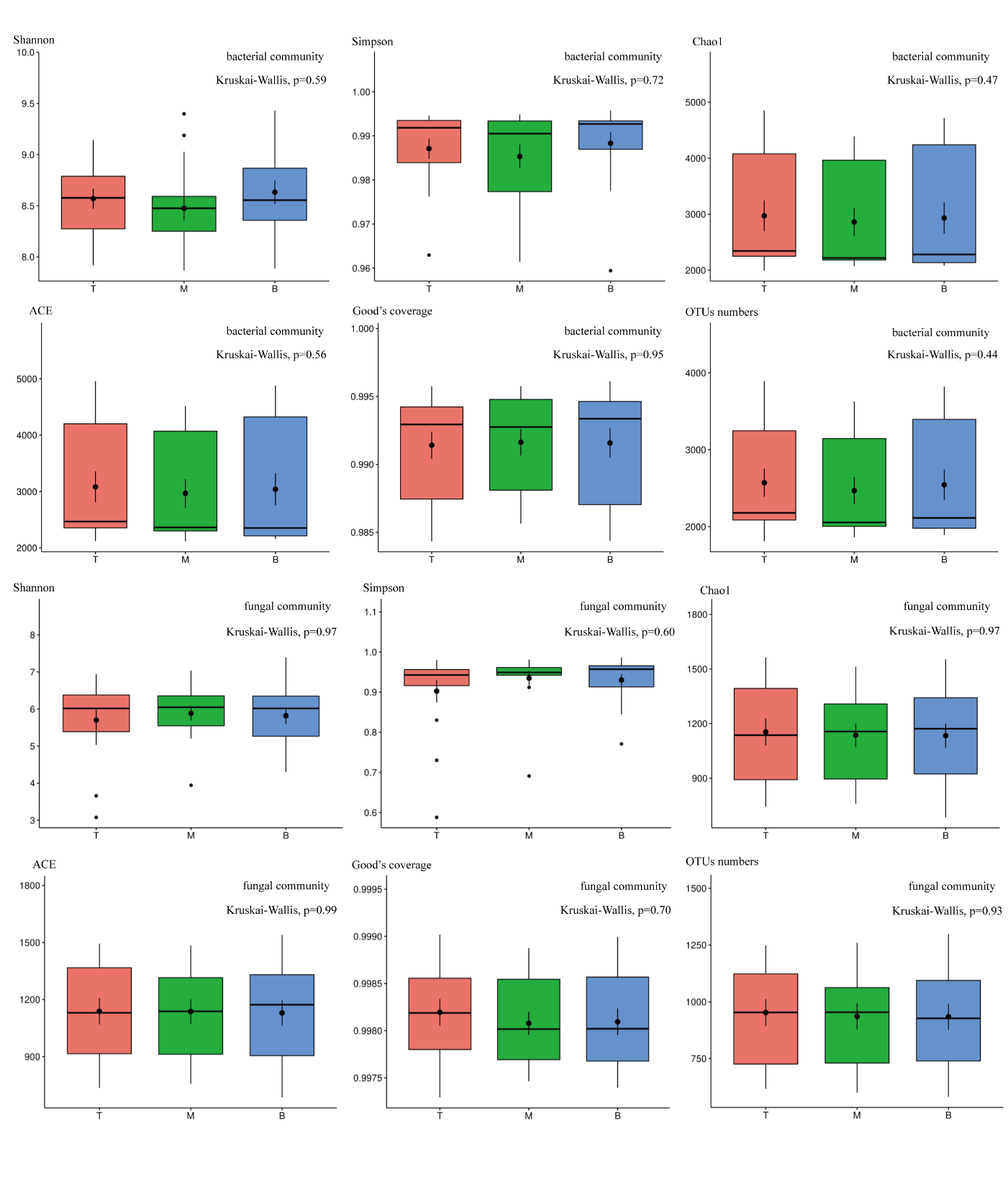


**Fig. S2** Test for differences of Alpha diversity index and OTUs numbers of microorganisms in different layers. T=0-20cm, middle=20-40cm, bottom=40-60cm layer, separately. Each Alpha diversity index showed no significant difference.


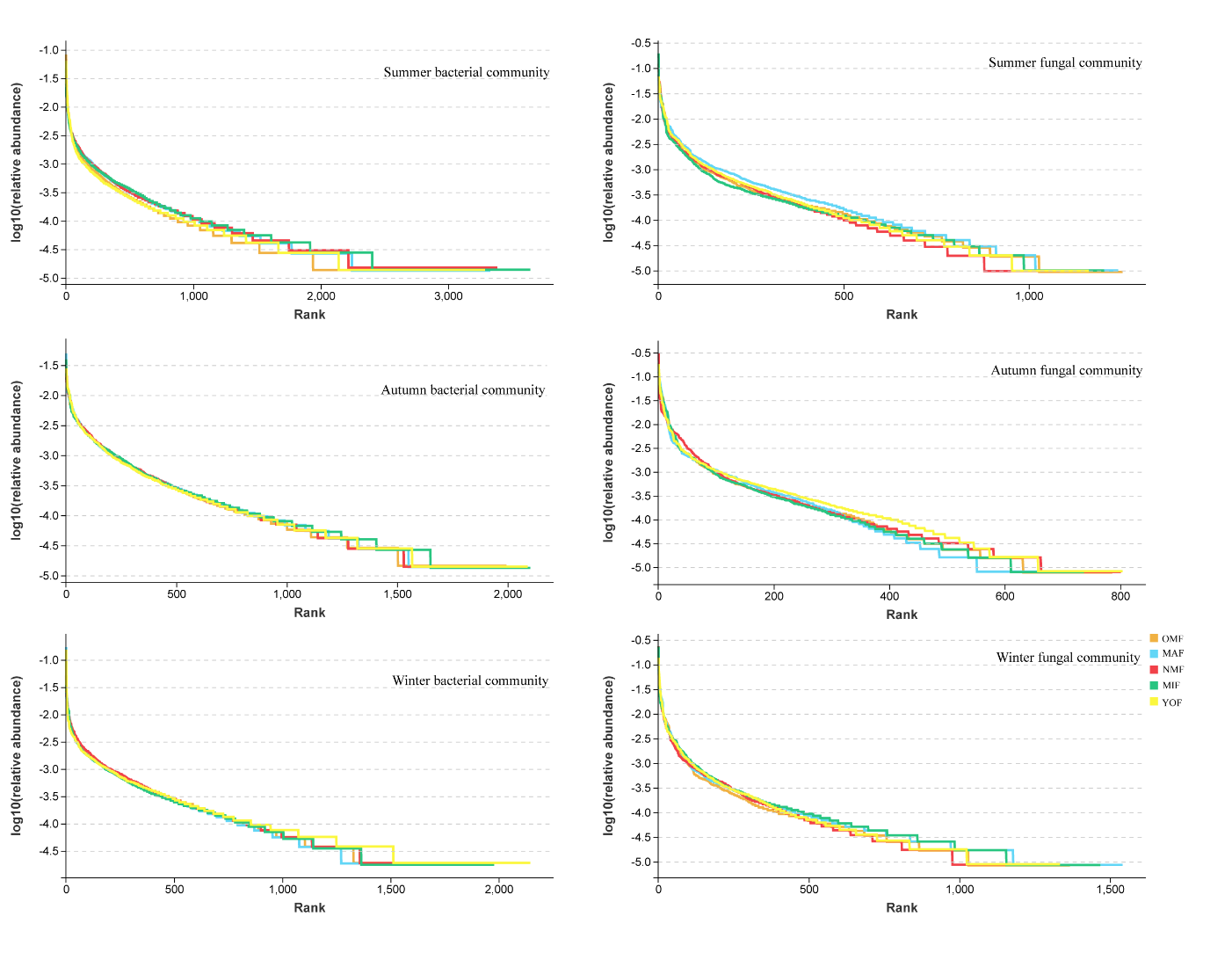


**Fig. S3** Rank Abundance curves of the microbial community in different stages and seasons. (OMF = overmature forest, MAF = mature forest, NMF = near mature forest, MIF = middle-aged forest, YOF = young forest)

**
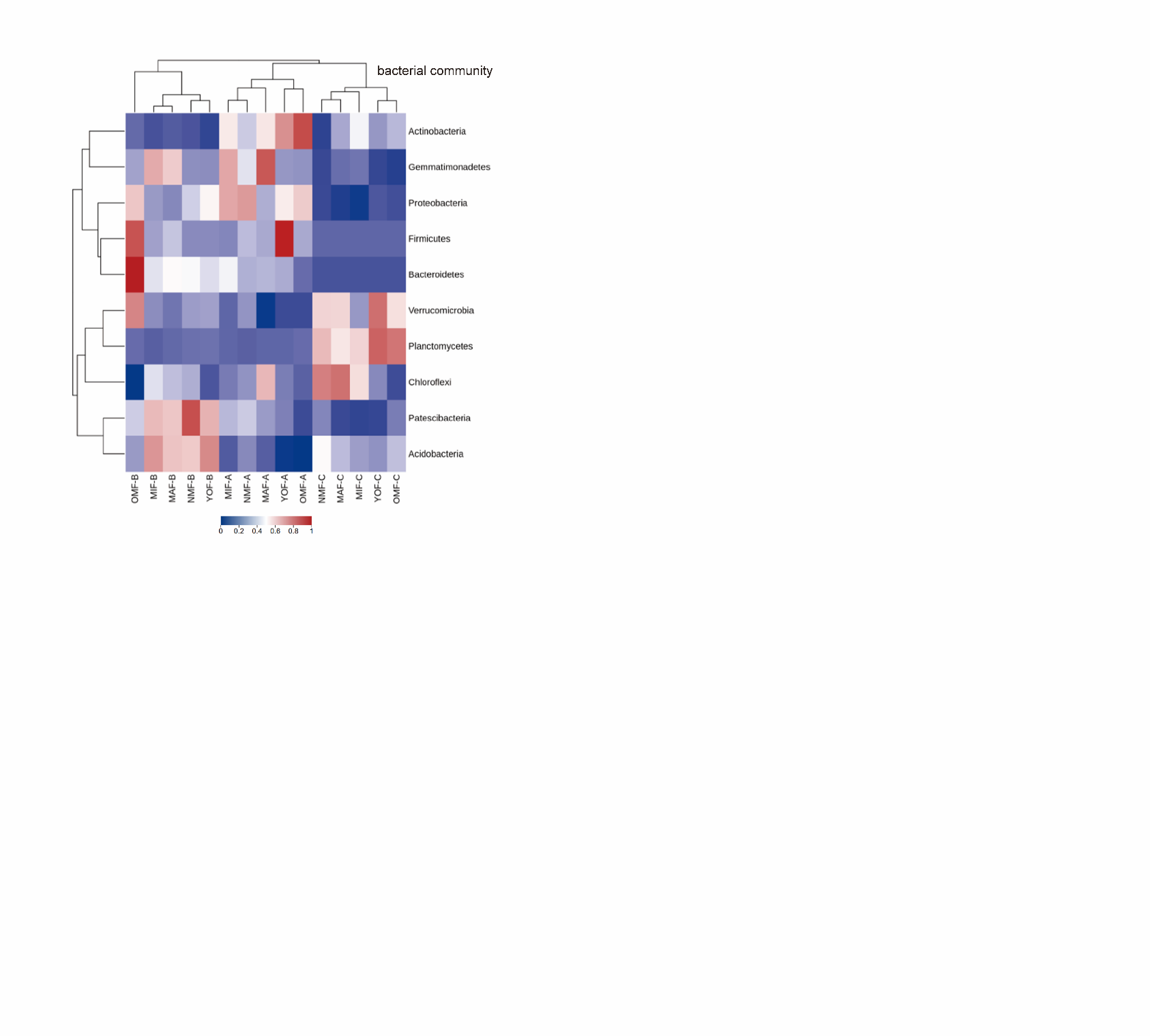
**

**Fig. S4** Heat map of soil bacterial communities in different forest age stands in different seasons at phylum level (OMF = overmature forest, MAF = mature forest, NMF = near mature forest, MIF = middle-aged forest, YOF = young forest; A = summer, B = autumn, C = winter)
